# The evolution of azole resistance through a reduced spore dormancy pathway associated with loss of SUMOylation function in *Fusarium graminearum*

**DOI:** 10.1128/aem.02342-25

**Published:** 2026-04-17

**Authors:** Kelsey Wog, Aruni S. Sumanarathne, Aleeza C. Gerstein, Matthew G. Bakker

**Affiliations:** 1 Department of Microbiology, University of Manitoba 468335, Winnipeg, Canada; 2 Department of Statistics, University of Manitoba 192323, Winnipeg, Canada; The University of Arizona, Tucson, Arizona, USA

**Keywords:** SUMO, demethylation inhibitor (DMI), fungicide resistance, fusarium head blight, Fusarium

## Abstract

*Fusarium graminearum* is a major pathogen of wheat and barley, causing fusarium head blight (FHB) and contaminating grain with harmful mycotoxins. Azole fungicides (demethylation inhibitors) are among the key tools available for managing *F. graminearum* infections. In an attempt to characterize how resistance to azole fungicides arises, we performed an experimental evolution study that imposed selection by exposing *F. graminearum* to increasing concentrations of prothioconazole (PTZ), tebuconazole (TBF), a combination of both fungicides (CMB), or a no-drug control. All evolved lineages exceeded the ancestral minimum inhibitory concentration before strain extinction, retained normal *in vitro* colony growth in the absence of drugs, and were able to successfully infect a fusarium head blight-susceptible wheat cultivar. However, most lineages lost their apparent resistance improvements after being revived from preservation at −80°C. One lineage (TBF1), however, showed stable enhanced resistance to tebuconazole, which was accompanied by a phenotype of precocious germination of macroconidia. Genomic analyses indicated no change in the *cyp51* genes in any lineage but identified a single base insertion resulting in a premature stop codon in the *aos1* gene (involved in SUMOylation) in the TBF1 genome. We created *aos1* gene deletion strains, which phenocopied TBF1 for both tebuconazole resistance and altered macroconidial germination. This work suggests that adjustments to spore germination processes may influence sensitivity toward fungicides and also highlights the role of SUMOylation in spore dormancy.

**IMPORTANCE:**

*Fusarium graminearum* is a major crop pathogen that can acquire mutations over time to common fungicides. Historically, studies have focused their attention on a single gene, *cyp51*, as the primary cause of resistance. However, there may be other pathways to enhanced resistance. For example, changes in macroconidium germination rates and transitions between cell types offer a route to fungicide resistance that has not been adequately appreciated to date. This study highlights a novel pathway to azole resistance, providing new insights into how *F. graminearum* may circumvent chemical controls. Through laboratory evolution, a single base insertion arose within the *aos1* gene, which caused phenotypes of altered macroconidium dormancy and reduced fungicide sensitivity. This research highlights the importance of experimental approaches that remain open to surprising evolutionary innovations and unexpected resistance mechanisms.

## INTRODUCTION

*Fusarium graminearum* is a critical global fungal pathogen of agricultural crops, causing fusarium head blight (FHB) disease on small grain cereal crops like wheat and barley (1), as well as gibberella ear rot on corn (2) and various seedling root rots (3). Economic impacts of *F. graminearum* infections frequently amount to hundreds of millions of dollars in losses (4). Preventing losses owing to this pathogen is best accomplished through integrated strategies that combine crop rotation, residue management, the use of crop cultivars that carry genetic resistance toward the disease, and chemical fungicides (5). Fungicides provide the most effective and economical control when their use is timed with crop phenology and with environmental conditions that favor pathogen establishment (6).

The development of fungicide resistance within pathogen populations is a concern. Among the fungicides available to manage *F. graminearum*, those based on azole compounds have been preferred for their broad range activity and relatively low cost (7). Environmental azole exposure is increasing; in the United States, azole use increased 434% from 2006 to 2016 (8). Furthermore, azoles are systemic and target site-specific, two traits that increase the risk of resistance evolution. Systemic fungicides are transmitted throughout the plant, increasing the proportion of the fungal cell population that is exposed to selection pressure, while a site-specific mode of action means that there are likely to be mutations that have large impacts on azole-target interactions.

Azoles are part of the Fungicide Resistance Action Committee (FRAC) Group 3, sterol biosynthesis inhibitor class I: demethylation inhibitors (9) and are, therefore, often referred to as “DMI” fungicides. These fungicides target the enzyme lanosterol 14α-demethylase (Cyp51), which is a cytochrome P450 that functions in the pathway that synthesizes ergosterol, a critical component of the fungal cell membrane (10). By inhibiting the lanosterol 14α-demethylase enzyme, azoles cause an accumulation of methylated precursors to ergosterol, which changes the sterol composition of the cell membrane and imposes stress. Across different azole-based fungicides, the active ingredients interact with Cyp51 in slightly different ways. For example, tebuconazole (TBF), epoxiconazole, and triadimenol directly interact with the enzyme’s heme iron, inhibiting the binding of oxygen (10, 11). In contrast, prothioconazole (PTZ) does not directly interact with the heme iron but seems to inhibit the action of Cyp51 through an alternate mechanism (11). Differences in the precise chemical interaction between various azole compounds and their cellular target suggest that there may be different opportunities for mutations to enhance resistance across different fungicide active ingredients.

For *F. graminearum*, as in all fungal species that have been studied for resistance toward azoles, the most common mutations that have been reported fall within or influence the expression of *cyp51* genes or influence the intracellular concentration of azoles (e.g., through the upregulation of efflux pumps) (7, 12, 13). Fungi differ in the number of *cyp51* genes they possess; there is a single gene copy in fungi such as *Candida albicans* (where the gene is known as *ERG11*) (14) and *Cryptococcus neoformans* (15), while *Aspergillus fumigatus* has paralogous *cyp51A* and *cyp51B* genes (16) and *F. graminearum* has three paralogs: *cyp51A, cyp51B,* and *cyp51C* (17). In *F. graminearum*, *cyp51B* encodes the primary sterol 14α-demethylase, while *cyp51A* encodes an additional sterol 14α-demethylase and seems to play a large role in variation in azole sensitivity (17). Based on an inability to compensate for Cyp51 function in *Saccharomyces cerevisiae*, the *Fgcyp51C* gene appears to no longer function as a sterol 14α-demethylase (17). Changes in the promoter regions of the *cyp51* genes can influence the transcription rate and might reduce sensitivity to azoles by increasing the number of Cyp51 proteins (7). Previously reported mutations in *F. graminearum* conferring resistance to tebuconazole include D243N or G443S mutations in *cyp51A*, as well as Y137H or S169Y mutations in *cyp51B* (18, 19). Resistance to metconazole is conferred by D243N, G443S, or E103Q + V157L mutations in the *cyp51A* gene (20).

Even given this prior knowledge, the genetic basis of resistance toward antifungal compounds is dramatically understudied for agricultural pathogens, including *F. graminearum*. A recent comprehensive database of reported antifungal resistance mutations in fungi (FungAMR, housed at https://card.mcmaster.ca/fungamrhome) assessed over 500 publications across 208 antifungal compounds (12). There are only 39 entries for *Fusarium* across all species, compounds, and study types (12). By comparison, 4 species have over 1,000 entries, while an additional 13 species have more entries than *F. graminearum* (12). For the common azole fungicide active ingredients metconazole and tebuconazole, the database reports eight entries for *F. graminearum* from three studies, all of which highlight *cyp51A* and/or *cyp51B*; only a single study has recreated a target mutation in *F. graminearum* and demonstrated an effect on resistance (12, 19).

Antimicrobial resistance is most problematic if resistant mutants retain normal or improved fitness in the absence of drugs, i.e., if resistance-conferring mutations do not carry associated fitness costs that would hinder resistant strains from increasing in frequency within a population. Beyond the potential to lose critical management tools for protecting agricultural productivity, the development of resistance to fungicides due to environmental azole exposure also raises concerns about cross-resistance to structurally similar azole drugs that are used to treat human fungal infections. This concerning phenomenon has recently been observed in *A. fumigatus*, where the widespread use of azole fungicides in agriculture has directly led to the evolution of *A. fumigatus* strains that are resistant to the first-line azole treatments for invasive aspergillosis. This makes these infections much harder to treat and leads to poorer patient outcomes (21, 22). Agricultural fungicide formulations often include multiple components in an effort to decrease the propensity for resistance evolution by requiring mutations to multiple targets. Yet if resistance does arise in the field, this could have critical implications for human health as *Fusarium* species were recently named one of the high-priority group fungal pathogens by the World Health Organization (23).

Deepening our understanding of the pathways through which resistance might evolve is critical for maintaining the efficacy of agricultural fungicides. One approach has been to genotype the least sensitive (i.e., most resistant) individuals within a wild-type population (24, 25). However, this approach suffers from confounded differences in the genetic background among strains, making it difficult to identify a causal role for particular polymorphisms in resistance (26). Association studies do sometimes reveal interesting mechanistic effects, such as a marker-trait association between sensitivity to tebuconazole and a gene whose product stabilizes membrane sterols (27). However, even nonsynonymous variants within well-characterized target genes may not be significantly associated with fungicide sensitivity in correlative studies (28). Experimental laboratory evolution is a complementary strategy that has the simplifying advantage of working within a single genetic background with the ability to precisely manipulate the environment to impose a single selective pressure, while holding other abiotic and biotic factors (such as temperature and nutrient availability) constant (29). Experimental evolution is a powerful system for studying the genetic basis of adaptation and evolution when paired with whole-genome DNA sequencing (30, 31). It does not require *a priori* assumptions about where beneficial mutations are likely to arise (e.g., in contrast to only resequencing *cyp51* genes to look for the genetic basis of drug resistance). Somewhat surprisingly, this powerful strategy has not been well exploited to understand the evolution of drug resistance in agricultural fungal pathogens (7, 12).

While there are multiple examples of *in vitro* selection for fungicide resistance in *Fusarium* spp., these have certainly not exhausted the insights that can be gained from this approach. Repeated culturing on a tebuconazole-containing medium enabled selection of several mutant strains of *F. graminearum* with enhanced resistance to tebuconazole (32); all changes were linked to mutations in *cyp51A* or *cyp51C* genes. Others have similarly selected for a tebuconazole-resistant strain of *F. culmorum* (33), although the genetic basis for enhanced resistance was not determined in this case. Repeatedly passaging *F. graminearum* on medium containing fludioxonil successfully led to the identification of point mutations conferring resistance to that fungicide (34). In some cases, mutagenesis has been used to enhance the rate at which variants can be screened; this has, for example, produced *F. graminearum* strains with enhanced resistance to benzimidazole (35) and to fluazinam (36). Selection on fungicide-containing media has revealed point mutations in the gene encoding for the target of pydiflumetofen, in *Fusarium solani* (37).

With the aim of identifying mutations that confer decreased sensitivity to azoles in *F. graminearum,* we performed an experimental evolution study, in which replicate lineages derived from a common ancestral strain were exposed to increasing concentrations of prothioconazole (PTZ), tebuconazole (TBF), or a combination of both azoles (CMB). We expected that the different active ingredients would select for different point mutations within the *cyp51* genes and that resistance-conferring mutations would arise less frequently in the presence of the azole combination. The terminal evolved strains were genotyped by whole-genome sequencing and phenotyped for growth rate, reproductive ability, and aggressiveness in causing fusarium head blight on wheat.

## RESULTS

### Experimental evolution

An ancestral strain of *F. graminearum* was evolved on solid medium in the presence of each of four drug exposure treatments: PTZ, TBF, a combination of both azoles in equal concentrations, or a DMSO no-drug control (i.e., exposure to solvent only; DMS). Drug exposure began at a concentration that reduced growth area but still permitted substantial growth when initiated from macroconidia. Actively growing mycelium was transferred every 5 days, while at every fifth transfer, the culture was passed through carboxymethyl cellulose (CMC) broth for macroconidia production. Conidia were aliquoted for cryopreservation and for spotting onto a fresh plate containing double the previous drug concentration (Supporting Data Fig. S1). The experiment was continued to extinction or when 40 transfers were reached (DMS control treatment only).

**Fig 1:**
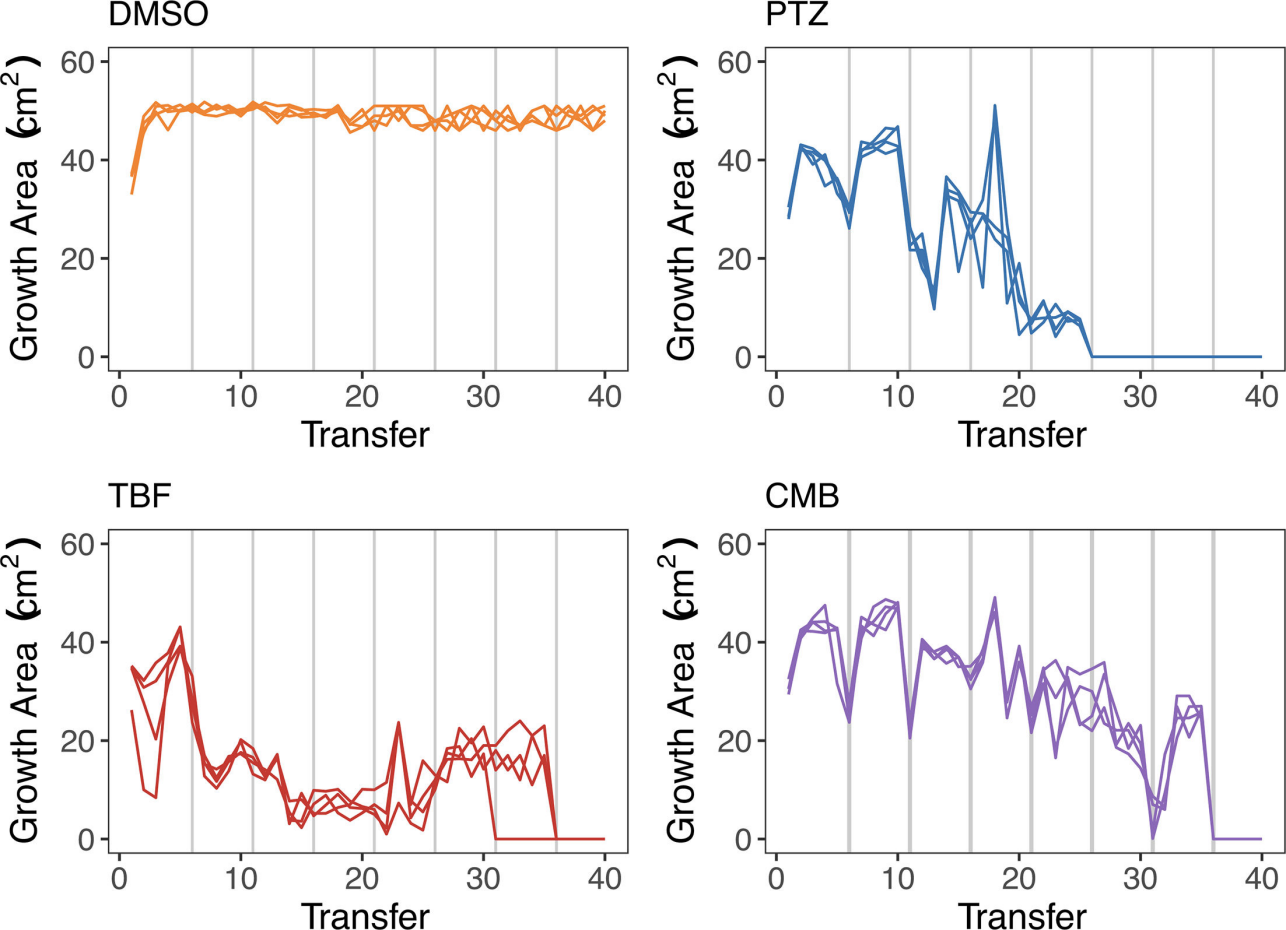
Evolution experiment. Each line represents one of four replicate lineages evolved in each treatment group. The experiment began at azole concentrations permitting substantial growth: 0.2 µM prothioconazole (“PTZ”; vs ancestral MIC of 3.2 µM); 0.8 µM tebuconazole (“TBF”; vs MIC of 6.4 µM); and 0.02 µM of each drug in combination (“CMB”; vs MIC of 0.08 µM PTZ + 0.02 µM TBF). The drug concentration in each treatment group was doubled at every fifth transfer (vertical lines). The first transfer at each new concentration was initiated from macroconidia, with the following four transfers initiated from a mycelium plug taken from the leading edge of the growth front. The growth area was measured 5 days after each transfer.

The four lineages evolved in the drug-free DMS control treatment exhibited consistent and steady growth throughout the experiment, with no substantial changes in growth area. In contrast, the lineages that were evolved to azoles displayed fluctuating growth patterns. Declines in growth area were observed at each increase in azole concentration (Fig. 1), which also corresponded to the use of macroconidia, instead of mycelia, as the inoculum. All drug-exposed lineages grew at concentrations exceeding the ancestral MIC. The point of extinction differed depending on the drug: PTZ lineages were extinct by transfer 25 (6.4 µM), while TBF and CMB lineages were extinct by transfer 35 (102.4 µM and 2.56 µM, respectively). We refer to the terminal population from each replicate lineage as the “evolved strain.”

### Acquired resistance was generally unstable

**Fig 2:**
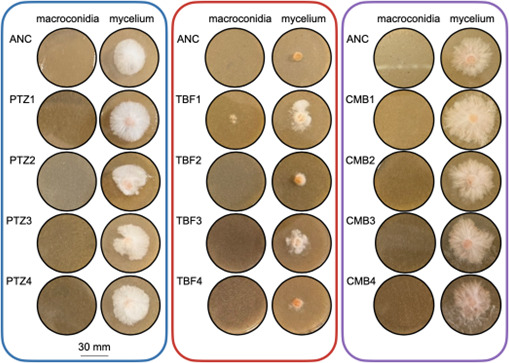
Growth at the terminal drug concentration in the evolved strains. Ancestral and evolved strains were grown from either macroconidia or a mycelial plug after revival from preservation at −80°C. The drug concentration on each plate is the maximum concentration at which growth was observed during the evolution experiment, as appropriate for each evolved strain.

The evolved strains were revived from −80°C freezer stocks and re-evaluated for their azole resistance. Surprisingly, when plates were inoculated with macroconidia, only the evolved TBF1 strain was able to grow at the highest (terminal) drug concentration from which a conidial suspension was preserved (i.e., the last drug concentration before lineage extinction) (Fig. 2). When inoculated as the mycelium, only evolved TBF1 and TBF3 strains grew better than the ancestral strain (Fig. 2). Thus, the enhanced growth above the ancestral azole MICs observed during the evolution experiment was lost in the majority of evolved strains upon revival from preservation in glycerol at −80°C.

### Most evolved strains were phenotypically similar to the ancestral strain

**Fig 3:**
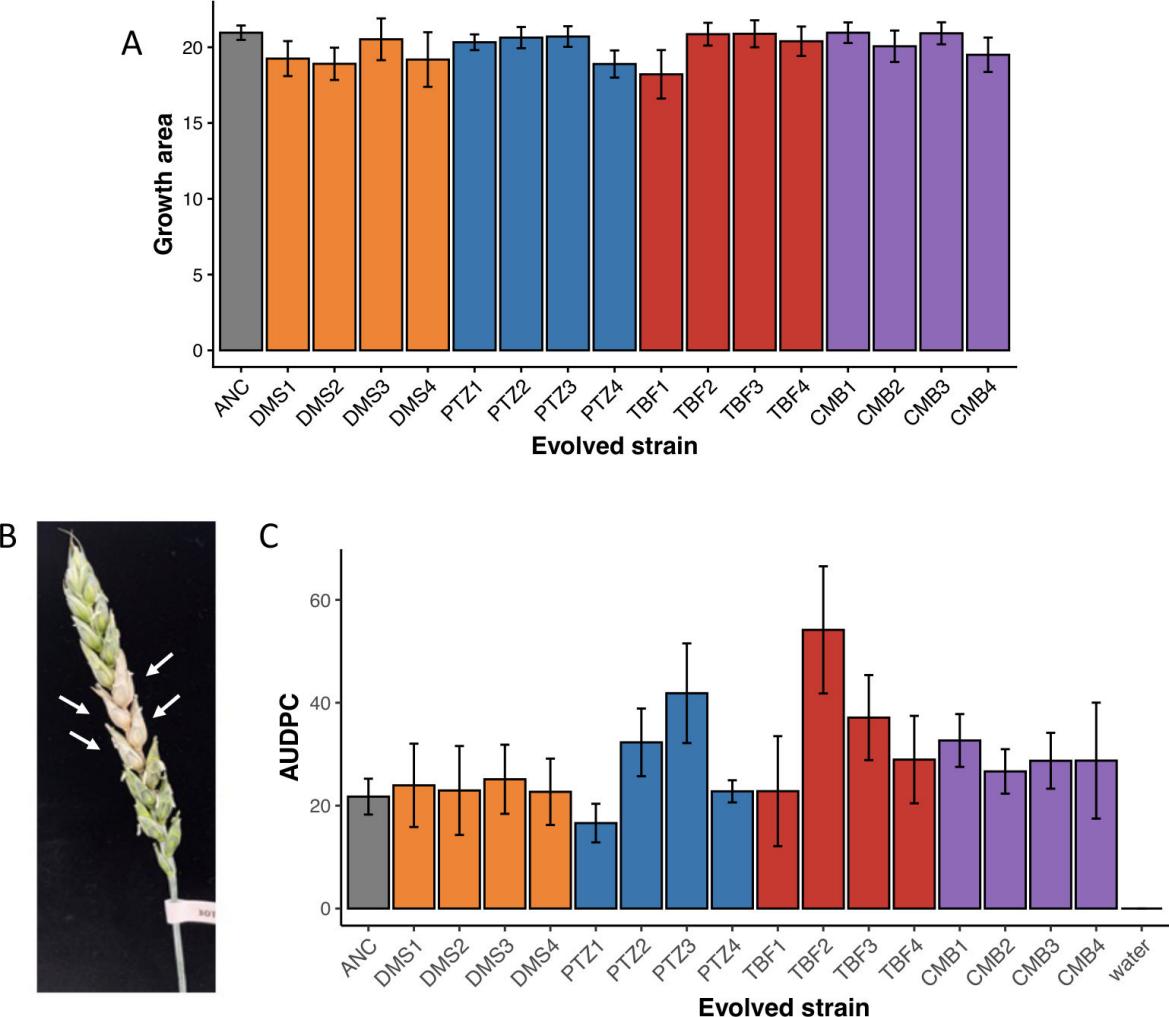
Evolved strain phenotypes in the absence of drug. (**A**) Colony growth area for evolved strains grown on azole-free V8 agar after 72 h, started from a mycelium plug. (**B**) A typical diseased wheat head from the experiment, showing four symptomatic spikelets (arrows). (**C**) The area under the disease progress curve (AUDPC), based on counts of symptomatic spikelets at days 4, 7, and 10 post-inoculation. The mean ± SE is shown.

There were no obvious costs to the evolved strains in the absence of drug. Growth after 72 h on azole-free V8 agar was not impacted for any of the evolved strains (Fig. 3A; one-factor ANOVA, F_16, 34_ = 0.745, *P* = 0.73), and all evolved strains successfully formed perithecia on carrot agar (data not shown). In an *in planta* greenhouse disease assay, all evolved strains retained the ancestral level of virulence, as indicated by the development of fusarium head blight symptoms on a susceptible variety of wheat (Fig. 3B, representative disease spikelet; Fig. 3C, area under the disease progress curve [AUDPC]; ANOVA: F_16, 163_ = 1.514, *P* = 0.10).

**Fig 4:**
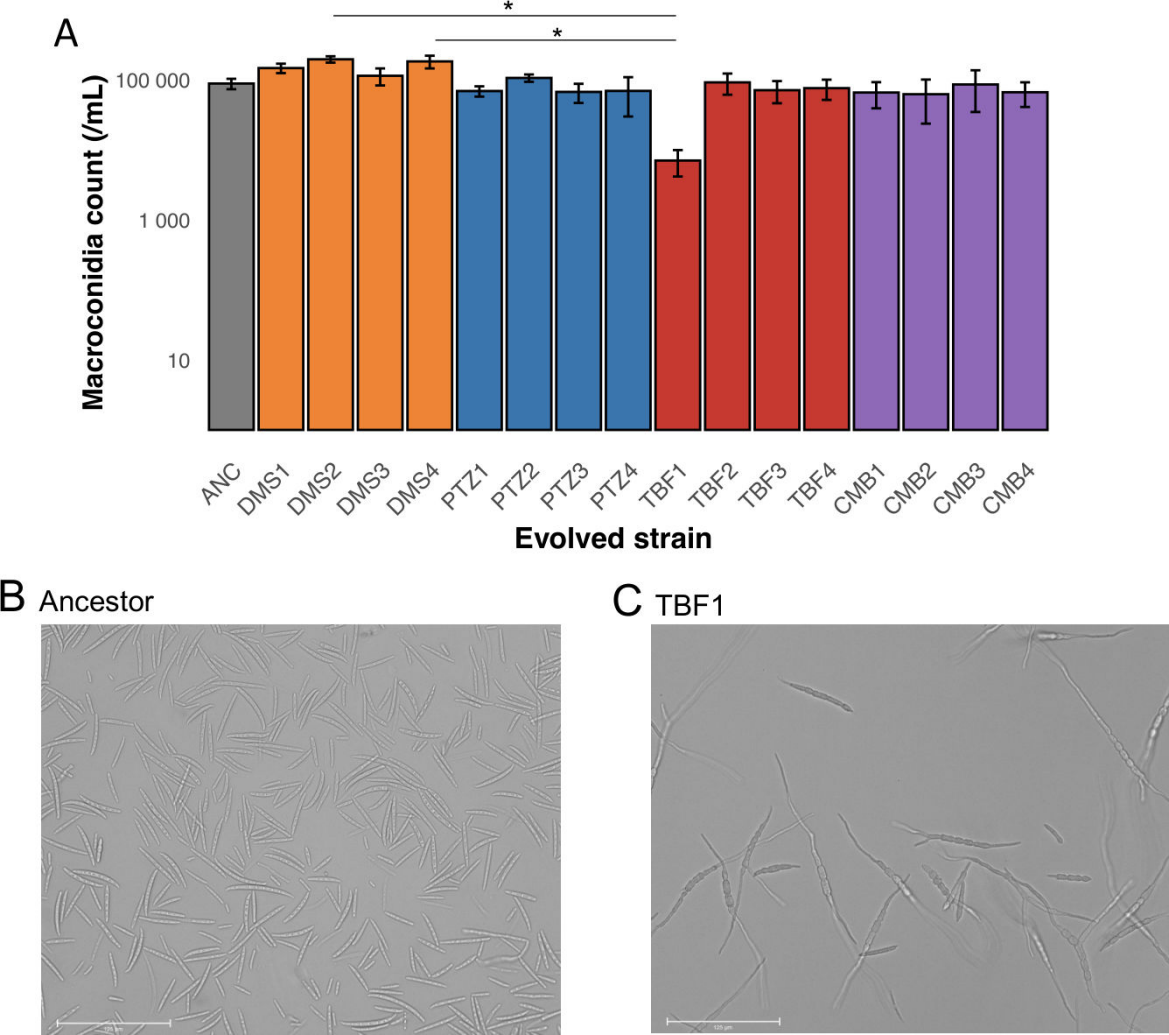
Macroconidia production of evolved strains. (**A**) Production of macroconidia by the ancestral and evolved strains in CMC broth. The bar height indicates the mean of three replicate flasks per strain, and error bars indicate the standard error. The two statistically significant strain comparisons, as determined by a Tukey test, are indicated by stars. Note that the statistical analysis of macroconidium counts was conducted on the untransformed counts, while the data are presented here on a log_10_ scale for ease of visualization. (**B**) Macroconidia from the ancestral strain and (**C**) germinating macroconidia from evolved strain TBF1. Macroconidia were suspended in sterile water at 4°C for 48 h and then photographed.

Evolved strain identity was, however, a significant factor in macroconidia production (Fig. 4; ANOVA: F_16, 34_ = 2.746, *P* = 0.007). A post hoc Tukey test indicated that the TBF1 evolved strain had significantly lower macroconidium counts than evolved strains DMS2 and DMS4 (*P* < 0.001 for those contrasts; *P* > 0.10 for all others). Visual evaluation of the TBF1 macroconidia indicated that they did not exhibit dormancy; unlike most macroconidia of the ancestral strain and of other evolved lineages, which remained dormant under cold, low-nutrient conditions (Fig. 4B), TBF1 macroconidia began to swell and germinate immediately upon formation (Fig. 4C). This early germination phenotype was unique to TBF1 among all lineages.

### Few high-impact variants arose during evolution

We performed short-read whole-genome sequencing of each of the 16 evolved strains. A total of 18 high-confidence genetic variants were identified across the set of 16 evolved strains, with the number of variants per strain ranging from 0 to 3 (mean 1.125; Table 1). Of these 18 variants, 11 were within genes: 5 were nonsynonymous (3 single-amino acid substitutions, 1 in-frame insertion of 4 additional amino acids, and 1 introduced premature stop codon), while 6 were synonymous (including 1 within a 5′ untranslated region). The remaining 7 were intergenic (201 to 1,628 bases upstream or downstream of the nearest gene, 4 of which were a mutation found in common across all 4 PTZ-evolved strains). Surprisingly, no mutations were identified in any of the *cyp51* genes (*cyp51A, cyp51B, or cyp51C*) in any of the evolved strains. Read depth patterns across the genome did not suggest any full or partial aneuploidies in any of the evolved replicates (Supporting Data Fig. S3). A 500 kb region on the left arm of chromosome 3 was deleted in TBF3 (Supporting Data Fig. S3), a region that encompasses 366 predicted genes. Structural variant analysis also revealed an 18 kb deletion (Chromosome 4: 3304776–3322974) in our ancestral strain (therefore also in all evolved strains), relative to the reference sequence for this strain background.

**TABLE 1:** Variants detected at the terminal time point across all laboratory-evolved lineages.^*a*^

Strain	Location	Feature	Ref	Alt	Variant ratio	Effect
DMS1	Chr 4: 2595604	gene_8334; serine/threonine-protein kinase TEL1	G	A	1.00	Synonymous
DMS2	Chr 1: 7161868	gene_2473; hypothetical protein	C	T	0.67	Nonsynonymous (S190L)
DMS2	Chr 1: 8364872	gene_2924; Gti1/Pac2 family-domain containing protein	A	AAGTGTTGCGAGTGTTGCG	Uncertain	In the 5′ UTR; reference has six tandem repeats of "AGTGTTGCG"
DMS4	Chr 2: 8344068	gene_7145; homeodomain-like protein	G	A	1.00	Synonymous
DMS4	Chr 2: 9028661	201 base pairs upstream	G	A	0.98	Intergenic
PTZ1, PTZ2, PTZ3, and PTZ4	Chr 4: 6957462	554 base pairs downstream	G	A	1.00	Intergenic
PTZ2	Chr 1: 1189467	gene_444; DNA repair metallo-beta-lactamase-domain-containing protein	C	T	0.98	Nonsynonymous (S767C)
PTZ2	Chr 4: 4900817	gene_9177; hypothetical protein	G	A	1.00	In the 5′ UTR
PTZ4	Chr 2: 3996443	gene_5485; cytochrome P450	G	A	0.58	Synonymous
PTZ4	Chr 4: 1255611	274 base pairs upstream	G	A	0.59	Intergenic
TBF1^*a*^	Chr 4: 2746703	gene_8383; *aos1*	T	TA	1.00	Premature stop (S102*)
TBF2	Chr 4: 4387258	gene_8994; mitochondrial carrier domain-containing protein	C	T	0.99	Nonsynonymous (G199S)
TBF3	Chr 2: 2999559	1,628 base pairs upstream	G	A	1.00	Intergenic
TBF4	Chr 1: 3483233	gene_1217; hypothetical protein	G	GTCGAGGGATCGT	0.15	In-frame, four amino acid insertion (R1547RDRSR)
CMB2	Chr 4: 6552285	gene_9732; hypothetical protein	G	A	0.96	Synonymous

^*a*^ This variant was also detected at transfers 20, 25, and 30.

### Early spore germination and azole resistance in the evolved TBF1 lineage were associated with a mutation in *aos1*

**Fig 5:**
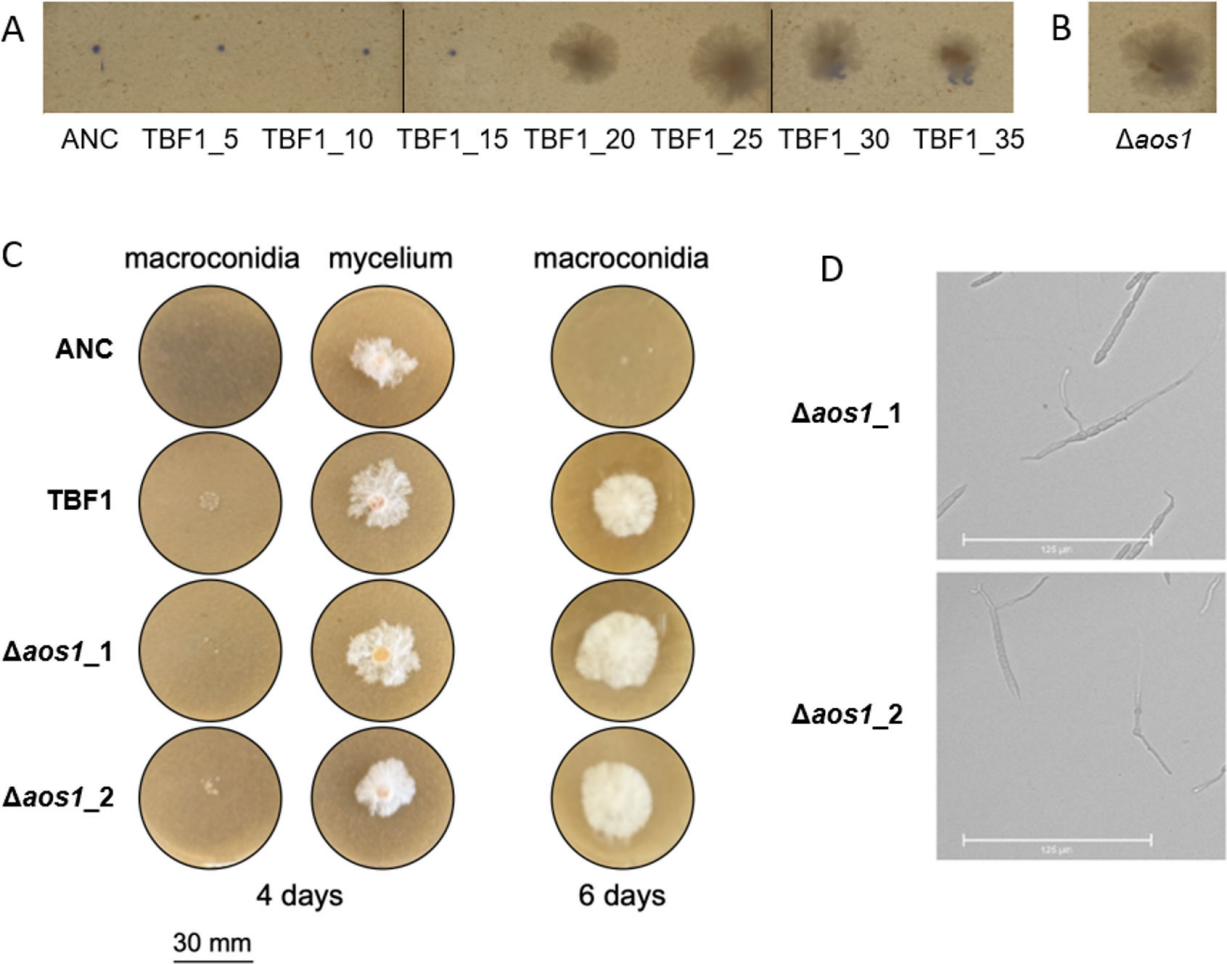
TBF1 and Δ*aos1* strain phenotyping. Macroconidia from (**A**) each preserved time point in the TBF1 lineage and (**B**) the Δ*aos1*_1 knock-out strain were inoculated on V8 agar with 20 µM tebuconazole and incubated for 4 days at 28°C. (**C**) Macroconidial and mycelial growth in the presence of 20 µM tebuconazole. (**D**) Macroconidia from Δ*aos1*_1 and Δ*aos1*_2 mutants were suspended in sterile water at 4°C for 48 h and then photographed.

In the TBF1 lineage, a single base insertion that caused a premature stop codon at amino acid S102 in the gene *aos1* (“activation of *smt3*”; gene ID: FGSG_05561; Mycocosm protein ID: 538,900) was identified. To determine at which point in the evolutionary progression the TBF1 lineage acquired its stable enhanced resistance, we revived the preserved stocks from throughout the lineage and tested their growth at 20 µM tebuconazole. The growth phenotype was identical to that of the final evolved strain from the 20th transfer to the terminal time point (Fig. 5A). Genomic DNA was isolated and whole-genome-sequenced from all preserved TBF1 time points. We found an exact correspondence between the phenotypic pattern of reduced sensitivity to TBF and the presence of the *aos1* mutation (i.e., the single base insertion was absent at the 15th transfer but present at the 20th transfer and all subsequent time points).

To confirm the phenotypic effect of this mutation, we engineered a Δ*aos1* deletion in the ancestral strain. Successful deletion of the *aos1* gene and insertion of a single copy of the hygromycin resistance gene were confirmed through qPCR and Sanger sequencing. Two separate knockout strains (Δ*aos1*_1 and Δ*aos1*_2) phenocopied the evolved TBF1 lineage for tebuconazole resistance (Fig. 5B) and for macroconidium phenotype (Fig. 5C and D). Thus, the observed mutation is responsible for both observed phenotypes through gene loss-of-function.

## DISCUSSION

The increasing use of fungicides, coupled with observations in some jurisdictions of increasing antimicrobial resistance in agricultural pathogens, including *F. graminearum* (8), underscores the critical need to better understand the dynamics of fungicide-driven evolution in populations of fungal pathogens. To study this directly, we evolved replicate lineages of *F. graminearum* to increasing azole exposure. During the evolution experiment, all replicate lineages grew at azole concentrations that surpassed the corresponding ancestral MIC, demonstrating a response to the selective pressure imposed by azole exposure.

It was surprising that the acquired growth improvement above the MIC observed during the laboratory evolution experiment was lost after revival of strains from storage at −80°C, with the notable exception of the TBF1 lineage. This instability, coupled with the absence of significant, stable genetic alterations in known targeted genes in these lineages, suggests that nongenetic factors may have been involved. A characterization of the mechanism(s) causing transient increases in resistance warrants future study. For example, can DNA methylation patterns or histone modifications produce an upregulation of responses to azole stresses? And are such regulatory effects reset during cryopreservation and resuscitation? Future experiments should include a profile of epigenetic markers prior to cryopreservation, such that changes in structural regulation of gene expression associated with long-term storage of evolved strains can be assessed. While there is little information yet available on the effects of cryopreservation on post-revival gene expression in fungi, studies in other systems have demonstrated that changes in DNA methylation and histone modification can result from cryopreservation (38–40). Epigenetic mechanisms are increasingly recognized as key regulators of fungal biology, influencing development, phenotypic plasticity, and the acquisition of antifungal resistance (41–45).

The phenomenon by which exposure to a stress leads to an enhanced response upon subsequent exposure to related stressors is referred to as “priming” (46, 47). A recent study reported evidence for priming in *F. graminearum* in response to tebuconazole and metconazole; conidia exposed to a partially inhibitory concentration of fungicides exhibited significantly higher germination rates and longer hyphal lengths when subsequently exposed to a doubled fungicide concentration, compared to unprimed conidia (48). The priming effect was retained for up to 30 days but was not stably heritable once the fungicide stress was removed; the effect was linked to overexpression of the *cyp51* genes, with regulatory influence by the transcription factor FgSR (48), which is itself regulated through SUMOylation (49). This example indicates that acclimation to higher fungicide exposures may arise through diverse interactions with gene regulatory networks. Future work should aim to describe specific mechanisms by which this occurs, distinguishing among categories of gene regulation and elaborating their implications for the stability and heritability of traits that impact sensitivity toward fungicides.

The observed transient nature of growth improvement under persistent azole exposure in most of our evolved *F. graminearum* lineages is very similar to observations from a recently published experimental evolution study involving *Candida albicans* and the broad-spectrum antimicrobial boric acid that used a similar experimental framework, yet evolved replicate lines growing as yeast in liquid culture (50). Given the extensive biological and biochemical differences between these two species and drugs, this suggests that unstable, nonmutational changes may be a common phenomenon enabling fungi to grow under high drug exposure when drug selective pressure is applied in an increasing fashion. Further work is needed to deeply characterize the interplay between adaptation and epigenetic or physiological acclimation under different drug dosing regimens in diverse species.

In contrast to the other evolved strains, TBF1 presented a stable, heritable phenotype of decreased azole sensitivity, particularly when macroconidia were exposed to the azoles. The evolved TBF1 strain was also the only phenotypic outlier for the other traits we examined, exhibiting altered macroconidium dormancy. Both phenotypes were linked to a nonsense mutation in *aos1*. Aos1 is involved in SUMOylation (SUMO = small ubiquitin-like modifier) as a component of the heterodimeric E1 activating enzyme, which acts early in the SUMOylation pathway (51). SUMOylation is a process that produces post-translational modifications on a variety of proteins that are involved in diverse cellular processes. In *F. graminearum,* for example, SUMOylation influences the cellular component targeting of a transcription factor and influences the efficiency of pre-mRNA splicing (49).

Several prior studies have investigated SUMOylation in *F. graminearum* or other *Fusarium* species. It has been previously demonstrated that loss of *aos1* is sufficient to abolish the SUMOylation of proteins (49). Links have also been found between SUMOylation and transcription factors and genes that are involved in conidiogenesis (52). Interestingly, prior reports have shown a reduction in virulence for a Δ*aos1* strain (49) and for a Δ*smt3* strain, which cannot produce the actual SUMO protein tag (52). By contrast, we did not observe a virulence defect in TBF1. Discrepancies with these studies may be related to differences in the genetic background of the *F. graminearum* strain in which the gene deletion was performed and/or to differences between the wheat varieties that were used in the disease assay. In one of these prior reports, a Δ*smt3* strain was also found to have no change in tebuconazole sensitivity or in the transcription of *cyp51A* and *cyp51B*. However, it is notable that this study used actively growing mycelium in their azole sensitivity assay (49); in our study, it was only when we exposed macroconidia to azoles that the resistance-conferring effect of the loss of SUMOylation function became evident. We might also expect that phenotypes may differ for Δ*aos1* vs Δ*smt3* strains; it has also been shown (in *F. oxysporum*) that phenotypic effects can differ depending on which component of the SUMOylation pathway is lost (53). Gene expression analysis in *F. oxysporum* showed that the expression of SUMOylation-related genes (*aos1*, *smt3*, and *mms21*) was significantly elevated in germinating macroconidia compared to the mycelium, while Δ*smt3* mutants suffered low germination rates (53), which is contrary to the spontaneous and accelerated germination we observed in Δ*aos1 F. graminearum* strains. Thus, comparison with *F. oxysporum* highlights the role of SUMOylation in spore germination, while also suggesting that the specific role of SUMOylation differs among species. Deciphering the mechanisms for the different impacts of disruption of SUMOylation function across these two species is thus required to improve our understanding of fungal cell biology and of spore dormancy and spore germination processes.

Our data suggest that loss of SUMOylation may result in a lack of perception of or response to environmental cues that typically hold macroconidia in a dormant state. Wild-type *F. graminearum* macroconidia germinate under particular environmental conditions (i.e., moisture availability, temperature, and nutrient availability). When these conditions are not met (i.e., dry, cold, and lack of nutrients), the wild-type *F. graminearum* macroconidia remain dormant. In the case of the Δ*aos1* or the evolved TBF1 strain, however, macroconidia germinate regardless of the surrounding environment, including both stressful conditions (azole exposure) and conditions that typically stimulate production of macroconidia (e.g., growth in CMC broth). Additional research to understand the role that the *aos1* gene and SUMOylation play in macroconidial germination cues would be fruitful. A study on *F. graminearum* evaluated gene expression at 0, 2, 8, and 24 h post-germination and found over 5,000 genes being expressed at each time point (54), showing the complexity of the germination process and the number of genes that are involved in germination.

Interestingly, our data indicate a pronounced difference in the sensitivity of *F. graminearum* macroconidia vs mycelium to azoles; a lower concentration of tebuconazole or prothioconazole was required to inhibit macroconidial germination than to inhibit mycelial elongation. In another study with the fungicide pydiflumetofen, the opposite was seen, where the EC_50_ value to inhibit *F. graminearum* spore germination was just over double the amount needed to inhibit mycelial elongation (55). More work is needed to understand the interactions between fungicide exposure and spore germination as such effects have significant practical field implications. For example, fungicides that are stronger inhibitors of germination should be used preventatively before fungal pathogens establishment, while fungicides that are stronger inhibitors of mycelial elongation may be more effective after pathogen establishment.

Features of the design of our evolution experiment may have contributed to the preponderance of unstable phenotypic responses to azole stress. Our evolution experiment was conducted on solid media to simulate the growth of the fungus on a plant having only surface azole contact, for the ability to quantify growth rates easily throughout the duration of the experiment, and to be able to intentionally advance cells with apparent beneficial mutations (i.e., deliberately selecting colony sectors with faster growth). Several prior studies have also imposed selection toward fungicide resistance by adding the fungicide active ingredients into solid media (32–34). However, this approach may have inadvertently reduced the intensity of selection pressures on the fungal population. For example, mycelial growth on solid surfaces can allow portions of the fungal colony to grow upward or away from direct contact with the azole in the agar. This escape mechanism could dilute the selection pressure, therefore reducing the fixation rate of beneficial mutations. In contrast, many other adaptive laboratory evolution experiments have been conducted in liquid media, providing more uniform exposure to the selective agent (26, 50, 56). To avoid selecting for mutations that would have severe fitness costs in a natural setting and to make it more likely that heritable phenotypic responses were being observed, our evolving lineages were passed through a macroconidia production stage after every five transfers. This process may, for example, have purged any mutations that would have abolished macroconidia production capacity. However, our choice to remove the azole exposure during spore production certainly impacted the selective environment as a whole and may have led to the loss of some resistance-conferring mutations within the experimental populations.

It is important to consider the fitness of evolved strains that have mutations that confer antimicrobial resistance. In the case of the evolved TBF1 strain, mutation in the *aos1* gene provided reduced sensitivity through early spore germination, effectively switching the exposure to a scenario of actively growing mycelium, in which the MIC is higher. However, some degree of macroconidium dormancy is likely to be critical to survival through unfavorable environments or episodes in the field, such as winter freezing or periods of drought. Furthermore, physical displacement of spores, such as by wind or rain splash, is critical to effective dissemination of the pathogen to suitable infection courts (e.g., dispersing from crop debris at the soil surface to the flowering structures at the apex of the plant). Therefore, the specific mutation observed in our evolution experiment, loss of function of *aos1*, is unlikely to be maintained in wild populations. However, our results suggest that a route to fungicide resistance is available through alteration or fine-tuning of spore germination processes, an evolutionary trajectory that has rarely been considered in the context of antimicrobial resistance acquisition among fungal plant pathogens.

Fungicidal compounds kill the fungus, while fungistatic compounds only inhibit its growth and reproduction. The effect of a compound is often determined by its concentration. For example, an antifungal drug that is fungicidal at high concentrations may be fungistatic at lower concentrations. While a fungicidal action is ideal for complete disease control, many widely used fungicides, particularly azoles that target the ergosterol biosynthesis pathway through inhibition of the Cyp51 enzyme, are primarily fungistatic at the label application rates (57). This fungistatic mode of action provides a critical opportunity for pathogens to evolve and adapt. Instead of being eliminated, the fungal population is merely suppressed, allowing a subpopulation of cells to survive the selective pressure. These surviving cells may possess pre-existing mutations or develop new, often transient, physiological or epigenetic mechanisms that enable them to tolerate the fungicide. For example, increased expression of efflux pumps can provide protection by rapidly expelling drugs from cells, as in the case of *F. culmorum* adapted to tebuconazole (58). Future work should explore whether the potential for acclimation (i.e., transient phenotypic plasticity in response to azole pressure) paves the way for stable genetic resistance or whether it may actually reduce the spread of more stable genetic resistance; for example, acclimation among competing strains may remove the selective advantage for strains with resistance mutations. Meanwhile, the epigenetic state may be reset over the winter season such that within-season acclimation to azole exposure need not compound from season to season.

Our motivation for including a mixed-drug treatment was the hypothesis that simultaneous exposure to two different azoles, which interact with different regions of the same target protein, would impose a more stringent selective pressure and be more difficult to overcome through naturally arising mutation. In other systems, it has been shown that multidrug regimens are often more effective than single drugs at preventing resistance development in bacterial infections (59). This principle is also applied in agricultural fungicide formulations, with commercial products such as Prosaro Pro (Bayer Crop Science) including a combination of prothioconazole and tebuconazole. However, our experiment did not produce any mutations directly in the target protein on which both fungicides act, which limited our ability to explore this hypothesis. Thus, further study designed to specifically probe evolutionary responses to mixed-azole fungicides is needed.

In conclusion, through a laboratory evolution experiment, we identified that altering spore germination processes can provide a route to enhanced resistance toward azole fungicides. This is an underexplored mechanism conferring resistance. Monitoring and additional research will be required to determine whether this pathway is followed in wild populations. We also highlight the enhanced basal resistance of *F. graminearum* mycelium toward azoles, compared to macroconidia, a phenomenon for which we do not currently have an explanation and which is opposite to what has been observed for another fungicide. Finally, we have demonstrated that SUMOylation is required for spore dormancy and that truncation or loss of the gene *aos1* results in precocious germination of macroconidia of *F. graminearum*.

## MATERIALS AND METHODS

### Laboratory-directed evolution experiment

Selective pressure was applied to replicate lineages of *F. graminearum* strain DAOMC 233423 (synonym GZ3639; ref 60) through a series of transfers done on solid-state medium (with the intention of partially mimicking growth on plant surfaces) under four different treatment conditions: a fungicide-free control (DMS) that was exposed only to the DMSO solvent carrier that was used in the other treatments, PTZ, TBF, and prothioconazole + tebuconazole combined (equal concentrations of each compound). Stock solutions of each fungicide active ingredient were prepared in DMSO.

The base growth medium was V8 agar (per L: 200 mL V8 juice, 0.3 g yeast extract, and 3 g calcium carbonate), and plates were incubated at 28°C in the dark. The MICs for PTZ, TBF, and CMB exposures were determined through an experiment that measured colony growth area at 72 h, beginning from 1,000 macroconidia spotted at the center of a series of plates containing increasing drug concentrations; the MIC for the ancestral strain was determined to be 3.2 µM for prothioconazole, 6.4 µM for tebuconazole, and 0.08 µM prothioconazole + 0.02 µM tebuconazole for the combined treatment (Supporting Data Fig. S4). To enable sufficient growth to accrue mutations, the evolution experiment began with the drug concentrations that were below the MIC: 0.2 µM for prothioconazole, 0.8 µM for tebuconazole, and 0.02 µM for each of the drugs in the combined treatment (note that we chose to equalize the drug concentrations in the combination treatment, and the selective pressure exerted by each drug likely differed; Supporting Data Fig. S4). The DMS treatment (control) received a DMSO exposure that was equal to that of the other treatments.

The ancestral strain was used to establish replicate lineages under each selection regime (*n* = 4 lineages per treatment) by spotting 10,000 macroconidia (concentration determined by hemocytometer) at the center of each plate. After 5 days, plates were photographed to capture the colony area, and then the culture was transferred to a new dish via a plug collected from the leading edge of growth using the back end of a 1 mL pipette tip. For the 5th transfer at a given drug concentration (i.e., after 25 days of continuous growth at the same concentration), the plug was instead transferred into carboxymethyl cellulose broth (per L: 15 g medium-viscosity carboxymethylcellulose sodium salt, 1 g NH_4_NO_3_, 1 g KH_2_PO_4_, 0.5 g MgSO_4_·7H_2_O, and 1 g yeast extract) to produce macroconidia for preservation (−80°C, in 30% sterile glycerol) as a “fossil record” along the evolutionary path of each lineage. After passing through CMC broth for spore production, the azole concentration was doubled; at each step increase, 10,000 macroconidia were spotted in the center of each Petri dish. This sequence of 25 days of mycelial growth at a consistent fungicide exposure, followed by spore production and doubling of the fungicide concentration, continued until there was no visible growth after 5 days of incubation. For lineages that went extinct, further characterizations were based on the end point of the previous azole concentration (i.e., the final preservation stock in the series).

### Phenotyping of evolved strains

After storage in the freezer for longer than 30 days, the endpoints of each lineage (“evolved strains”) were revived onto azole-free V8 agar. The strains were incubated at 28°C for 72 h before the mycelium was transferred into 30 mL of CMC broth in a 125 mL conical flask and shaken at 200 rpm at 28°C for 72 h. The inoculated broth was strained through a 40 µm cell strainer into a centrifuge tube. The macroconidia were spun down and resuspended in sterile water. The concentration of macroconidia was determined using a hemocytometer.

#### Growth in the absence of drug

To check whether standard growth was impacted by evolution under azole pressure, colony diameters were assessed on a drug-free medium. To ensure an equal readiness to grow in all tests, macroconidia were spotted onto drug-free V8 agar, allowed to grow for 4 days, and then a plug from the leading edge of growth was transferred onto another drug-free plate for assessment of growth. Photographs were taken at 72 h, and the colony surface area (cm^2^) was calculated in ImageJ, setting the scale for each image by the diameter of the Petri dish. Measurements were taken from three biological replicates.

#### Growth in the presence of drug

To check whether the evolved strains (end points of each lineage, as well as time points along the TBF1 lineage) retained their acquired resistance after storage at −80°C, strains were revived on drug-free V8 agar. Plugs from the leading edge of growth, or concentration-adjusted macroconidia from drug-free CMC broth (as described elsewhere), were transferred to the center of new V8 agar plates containing an appropriate drug concentration (i.e., the highest concentration at which successful growth was observed during the evolution experiment).

#### FHB assay

Aggressiveness in causing FHB was assessed with an *in planta* greenhouse experiment. Growth medium was prepared by combining 100 g of fertilizer (Osmocote 14-14-14) per 20 L of potting mix (Sungro Sunshine Mix). Pots (4 L) were lined with a double layer of paper towel and filled to 5 cm from the top with the prepared soil mixture, lightly compacted. An FHB-susceptible cultivar of wheat, CDC Teal, was planted with four seeds per pot. Slow-release fertilizer (13-12-12 ACER NT 3–4 months + micros) was spread on top of each pot (18 g per pot). The plant was considered to be the experimental unit. Plants were thinned to three plants per pot, and pots were culled from the experiment if fewer than three plants emerged. Pots were watered when the soil appeared dry. Additional fertilizer (20-20-20 Peters Professional) was provided every 14 days (125 mg in 500 mL per pot) until spike formation, at which point, fertilization was discontinued. Within sets of 21 plants (seven pots), treatments were applied in randomized order, with one plant inoculated with each of the sixteen evolved strains, four plants inoculated with the ancestral strain, and one mock-inoculated control plant. Each evolved strain was inoculated onto 10 heads, each on a different plant. The inoculum consisted of macroconidia, prepared from CMC broth as described above, but resuspended in sterile water at a concentration of 1,000 macroconidia per 10 µL. After at least one wheat head on each plant began flowering, the middle spikelet of a flowering head was marked, and 10 µL of the appropriate inoculum was pipetted into a floret. Inoculated heads were sprayed with water and covered with a plastic bag to maintain a humid environment conducive to infection. The plastic bags were removed after 48 h. The number of visually symptomatic (bleached) spikelets was recorded 4, 7, and 10 days post-inoculation. Nineteen plants were omitted from data collection due to mechanical damage or the appearance of non-fusarium head blight disease symptoms.

#### Reproductive capacity

To assess the reproductive capacity, we observed the number and morphology of macroconidia by microscopy (Evos Cell Imaging System, ThermoFisher Scientific). We also tested the ability to produce perithecia through homothallic sexual reproduction via cultivation on carrot agar (boil 100 g of fresh organic carrots for 10 min in 250 mL of water, and blend until smooth; add 5 g of agar); each plate was inoculated at the center and incubated in the dark for 6 days, at which point, the mycelium was flattened and removed with 1 mL filter-sterilized 2.5% Tween 60 and a stick spreader. The plates were placed under 24 h fluorescent light for 10 days, repeating the scraping whenever the mycelium appeared to grow. Perithecia were examined using a dissecting microscope.

### Genotyping of evolved strains

We performed short-read whole-genome sequencing to genotype the evolved strains (end points of each lineage, as well as time points along the TBF1 lineage). The mycelium was harvested from deep Petri plate cultures on V8 agar, lyophilized, and ground to a powder (30 s, 3.1 m/s; OMNI International Bead Ruptor Elite). DNA was extracted using the DNeasy Plant Pro kit (Qiagen), followed by additional purification (Clean and Concentrate kit, ZYMO Research). DNA quality and concentration were quantified spectrophotometrically (NanoDrop One, Thermo Scientific). The concentration of DNA in each sample was normalized to 25 µg/µL, and extracts were sent to SeqCoast Genomics (Portsmouth, USA) for library preparation (DNA Prep tagmentation kit with unique dual indexes, Illumina) and sequencing (NextSeq2000 with 2 × 150 bp paired-end reads, Illumina). DRAGEN v4.2.7 was used to assess read quality, demultiplex, and trim adapter sequences.

The sequence reads were processed with Trimmomatic v0.39 (61) to remove low-quality bases from the leading and trailing ends of each read, with default parameters. Quality scores were assessed with FASTQC v0.12.1 and summarized with MultiQC v1.13 to ensure the trimming process had successfully improved the data quality (62). The reference genome accessed from Mycocosm (*F. graminearum* Z3639 v4.0; this is a synonymous name for the same strain as DAOMC 233423) was indexed using bwa v0.7.17 with the IS indexing algorithm. Using the Genome Analysis Toolkit (GATK) v4.4.0.0 (63), a sequence dictionary was created for the reference sequence, and the reference FASTA file was indexed using SamTools v1.20 faidx (64). The alignment of the trimmed reads to the reference was computed with bwa-mem (65). Trimmed reads were further processed using Picard Tools v2.27.4 (66); the alignment was sorted by genomic coordinates using SortSam, and alignment quality metrics were collected using CollectAlignmentSummaryMetrics. The coordinate-sorted SAM files were converted to Binary Alignment Map (BAM) files using Picard SamFormatConverter for more efficient computing use. Read group information was added to the BAM files using Picard AddOrReplaceReadGroups, the duplicate reads were identified and marked using Picard MarkDuplicates, and the mate-pair information was corrected using Picard FixMateInformation. The GATK Best Practices were used for variant calling; HaplotypeCaller, CombineGVCFs, GenotypeVCFs, VariantFiltration, and SelectVariants (67–69) were used to identify single-nucleotide polymorphisms (SNPs) and INDELs among all sequenced lines in the haploid mode, requiring a minimum read depth of 30 for variant identification. All flagged putative variants were manually verified against read mappings; nonconfident variants (for example, variants in regions that were repeat-masked in the reference genome and variants occurring in long homopolymeric runs) were filtered out from the VCF file to generate the final VCF file (70).

Across the sequenced strains, the average depth of coverage was 87×, with minimum coverage of 60× and maximum coverage of 105×. Coverage across the genome (i.e., the number of reads that mapped to each base pair) was determined from the BAM file using the genomecov function in bedtools (71). The results were visualized in R using a custom script. Read depth for the position line was scaled by the median coverage across chromosome 1 for each line. The CLC Genomics Workbench (Qiagen) was used to visualize read alignments and to search for structural variants.

### Gene knockout using CRISPR-Cas9

We used CRISPR-Cas9 gene editing to validate one mutation of interest, following published protocols (72, 73). Macroconidia (1 mL at 2 × 10^6^ macroconidia/mL) were inoculated in 50 mL of oxgall media (per L: 15 g oxgall, 10 g peptone, and 10 g glucose) and shaken at 200 rpm at 22°C. Germinated macroconidia (germ tubes were 2–3 times the length of the macroconidium) were trapped on a 0.45 µm mesh cell strainer, washed with 1.2 M KCl, and incubated in an enzyme cocktail (500 mg yatalase, 200 mg driselase, and 200 mg lysing enzyme in 20 mL of 1.2 M KCl; filter-sterilized) at 100 rpm and 28°C. Protoplasts were filtered through a 40 µm cell strainer, pelleted with gentle centrifugation (3,500 rpm, 5 min), washed 3 × with 25 mL 1.2 M KCl, and resuspended in STC buffer with DMSO (per 200 mL: 43.72 g sorbitol, 2 mL 1 M Tris-HCl pH 8, 1.47 g CaCl_2_·2H_2_O, and 6 mL DMSO; 2 × 10^7^ protoplasts/mL). Aliquots of suspended protoplasts were stored at −80°C.

CRISPR RNAs (crRNA; sequences in Supporting Data Table S1; Integrated DNA Technologies) were designed based on the flanking regions of the *aos1* gene, using CHOPCHOP (74). The target site was selected based on a high efficiency score, with GC content between 40 and 60, and no self-complementary regions.

The homologous repair template was prepared from the pRF-HU2 plasmid containing the hygromycin phosphotransferase gene *hph*. A forward primer was designed to amplify *hph*, while including a 5′ overhang corresponding to the 35 bases before the start codon of the *aos1* gene. Similarly, the reverse primer primed the 3′ end of the *hph* gene and included an overhang corresponding to the sequence immediately downstream of *aos1* (sequences in Supporting Data Table S1; ordered as Ultramer DNA Oligos from IDT). High-Fidelity Phusion Master Mix (Thermo) was used for amplification.

The Cas9 enzyme was purchased (Alt-R S.p. HiFi Cas9 Nuclease V3; IDT), and the Cas9-gRNA ribonucleoprotein (RNP) complex was assembled *in vitro* before being introduced to protoplasts. The RNP complex, protoplasts, 9 µg of homologous repair template, and 25 µL of PEG 4000 (per 100 mL: 60 g polyethylene glycol, 45 mL 1 M Tris-HCl, and 0.74 g CaCl_2_·2H_2_O) were combined and incubated at room temperature for 20 min. The volume was filled to 2 mL with STC buffer, then 2–3 mL of TB3 media (per L: 3 g yeast extract, 3 g casamino acids, and 200 g sucrose) was added, and the tube was incubated at 150 rpm and 25°C for 18–20 h. Regenerated protoplasts (300 µL suspension) were mixed with 20 mL of fresh molten TB3 containing 0.7% low-melting point agarose and 0.1 mg/mL hygromycin B.

PCR was used to confirm that the intended edit occurred in colonies that grew on the selection plates. DNA was extracted as described above from the evolved TBF1 strain and two CRISPR-edited strains (Δ*aos1*_1 and Δ*aos1*_2). PowerTrack SYBR Green Real-Time PCR Master Mix was used for qPCR, targeting three genes of interest: the *hph*, *aos1*, and a reference single-copy gene at a different locus, *tri6*. The qPCR was run with technical duplicates and a thermal cycling program consisting of 40 cycles of the following: 95°C for 15 s, 60°C for 1 min, and plate read. In addition to validating the absence of *aos1* and the presence of *hph* where expected, the copy number of the inserted hygromycin resistance gene was estimated as follows: gene copy number = 2^(Ct_^*^tri6^*^-Ct_^*^hph^*^)^.

### Macroconidium imaging

Macroconidia were produced as described above for the ancestral strain, the evolved TBF1 strain, and Δ*aos1*_1 and Δ*aos1*_2. Macroconidia were strained through a 40 µm cell strainer, washed twice with 30 mL sterile water, and counted with a hemocytometer. One hundred macroconidia were placed with 200 µL of sterile water per well in a 96-well plate. The plate was incubated at 4°C for 48 h and then imaged (Evos Cell Imaging System, ThermoFisher Scientific).

### Statistical analyses

Growth areas and macroconidium counts were analyzed with an analysis of variance (ANOVA) test with Tukey *post hoc* contrasts. For the plant experiment, counts of symptomatic spikelets were summarized using the area under the disease progress curve (AUDPC) method (R package agricolae [75]). The resulting AUDPC values were then analyzed by an ANOVA.

## Data Availability

Raw sequence data are available at NCBI as accession no. PRJNA1381695. All phenotypic data, SLURM job scripts for the variant calling pipeline, and R scripts required to reproduce statistical analyses and to generate figures are available at https://github.com/MicroStatsLab/Fgram_azoleExpEvol.

## SUPPLEMENTAL MATERIAL

Supplemental figures aem.02342-25-s0001.pdf Fig. S1 to S4.

Table S1 aem.02342-25-s0002.xlsx Oligos used in this work.

ASM does not own the copyrights to Supplemental Material that may be linked to, or accessed through, an article. The authors have granted ASM a non-exclusive, world-wide license to publish the Supplemental Material files. Please contact the corresponding author directly for reuse.
