# Supplementary material for: The evolution of azole resistance through a reduced spore dormancy pathway associated with loss of SUMOylation function in Fusarium graminearum
Source: Appl Environ Microbiol. 2026 Apr 17;92(5):e02342-25. doi: 10.1128/aem.02342-25 (PMC13188882; doi:10.1128/aem.02342-25)
Supplement: Supplemental figures — Fig. S1 to S4. [file aem.02342-25-s0001.pdf]

Wog K, Sumanarathne AS, Gerstein AC, Bakker MG. 2026. The evolution of azole resistance through a reduced spore dormancy pathway associated with loss of SUMOylation function in *Fusarium graminearum*. Applied & Environmental Microbiology.

Supporting Data

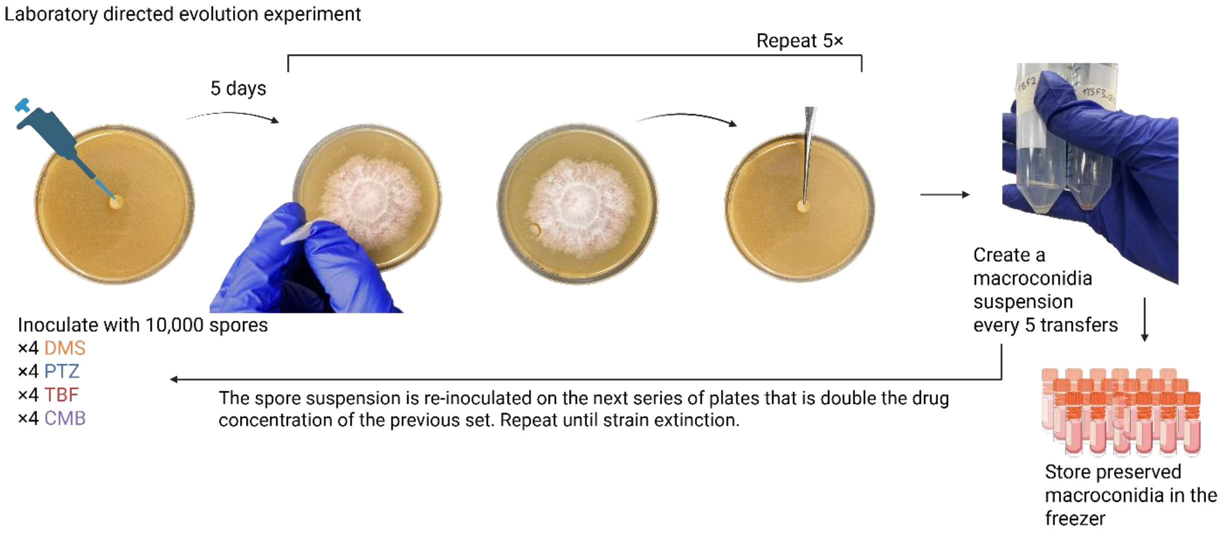

Supporting Data Figure S1 – A summary of the experimental procedures.

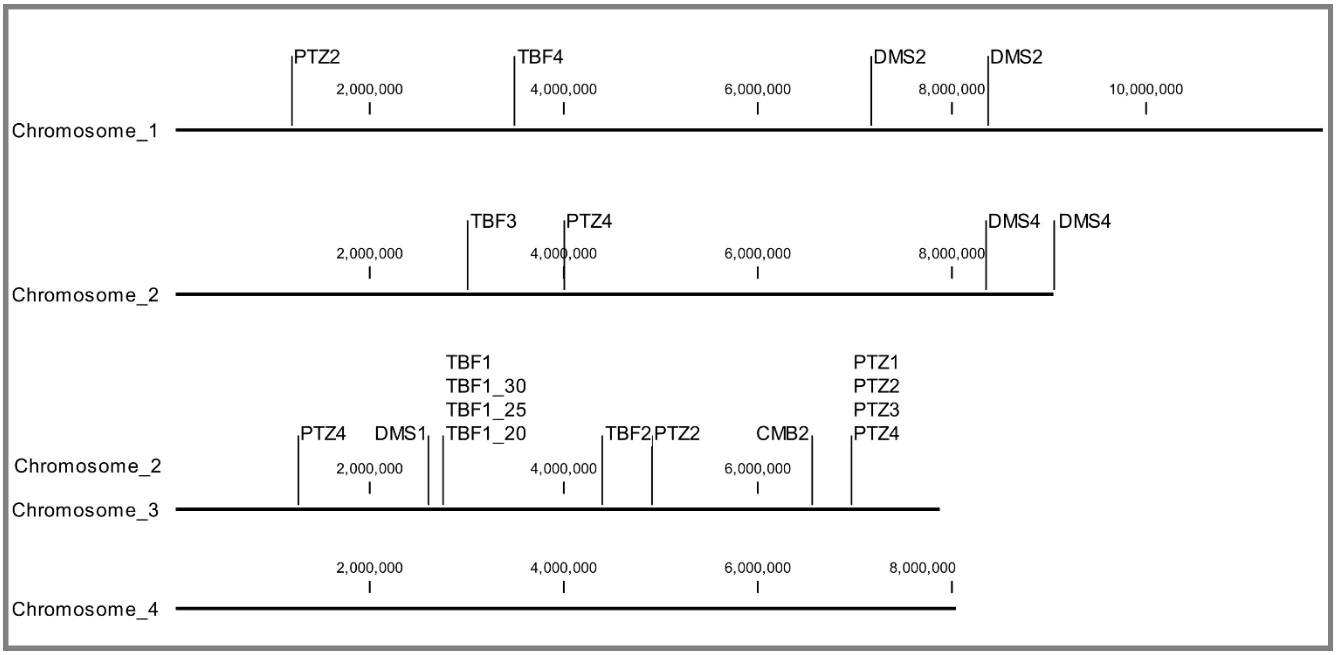

Supporting Data Figure S2 – The distribution of observed variants across the genome. Labels indicate the lineage in which the observed variant was observed.

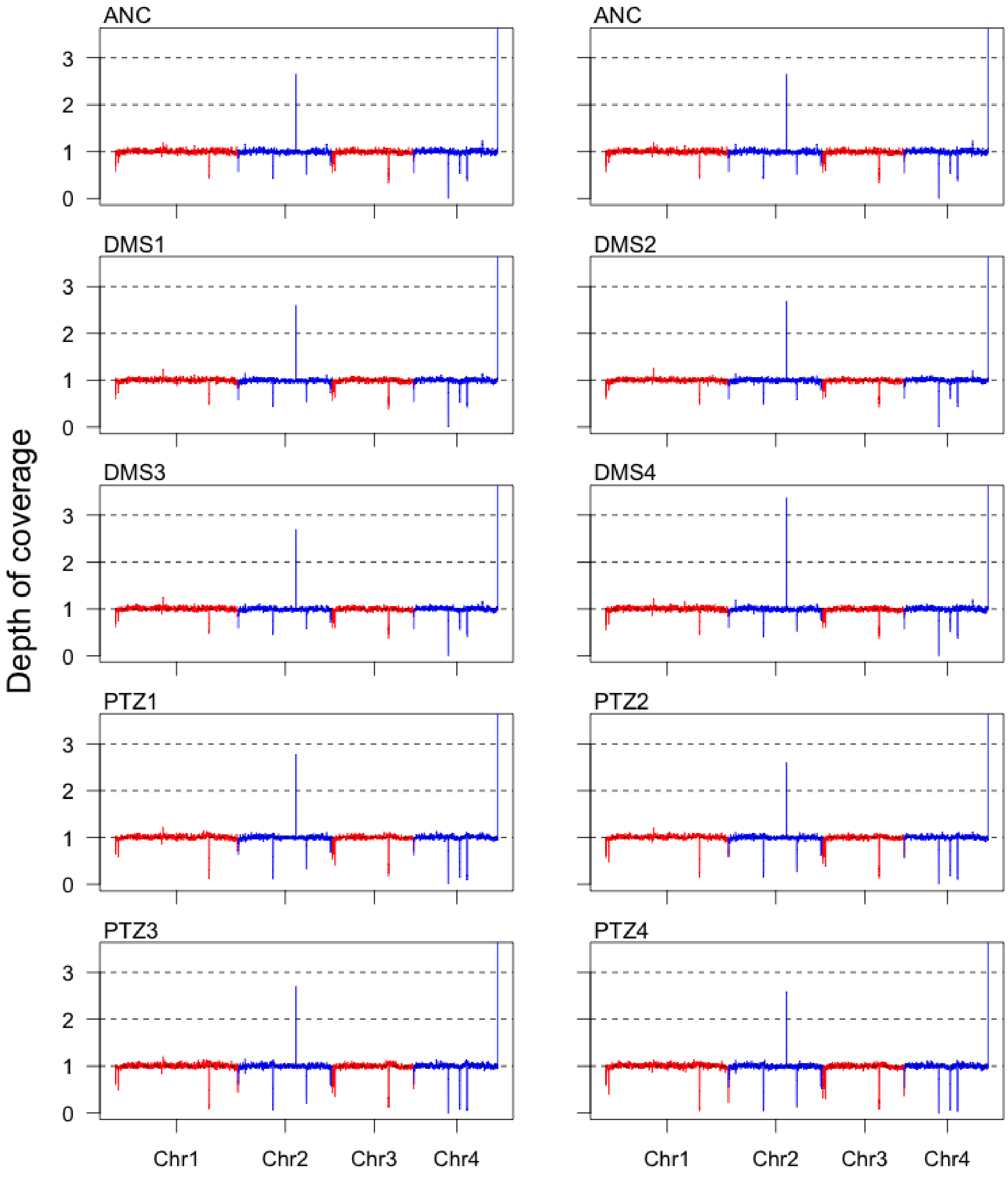
Depth of coverage
ANC
ANC
DMS1
DMS2
DMS3
DMS4
PTZ1
PTZ2
PTZ3
PTZ4
0
1
2
3
Chr1
Chr2
Chr3
Chr4

(continued)

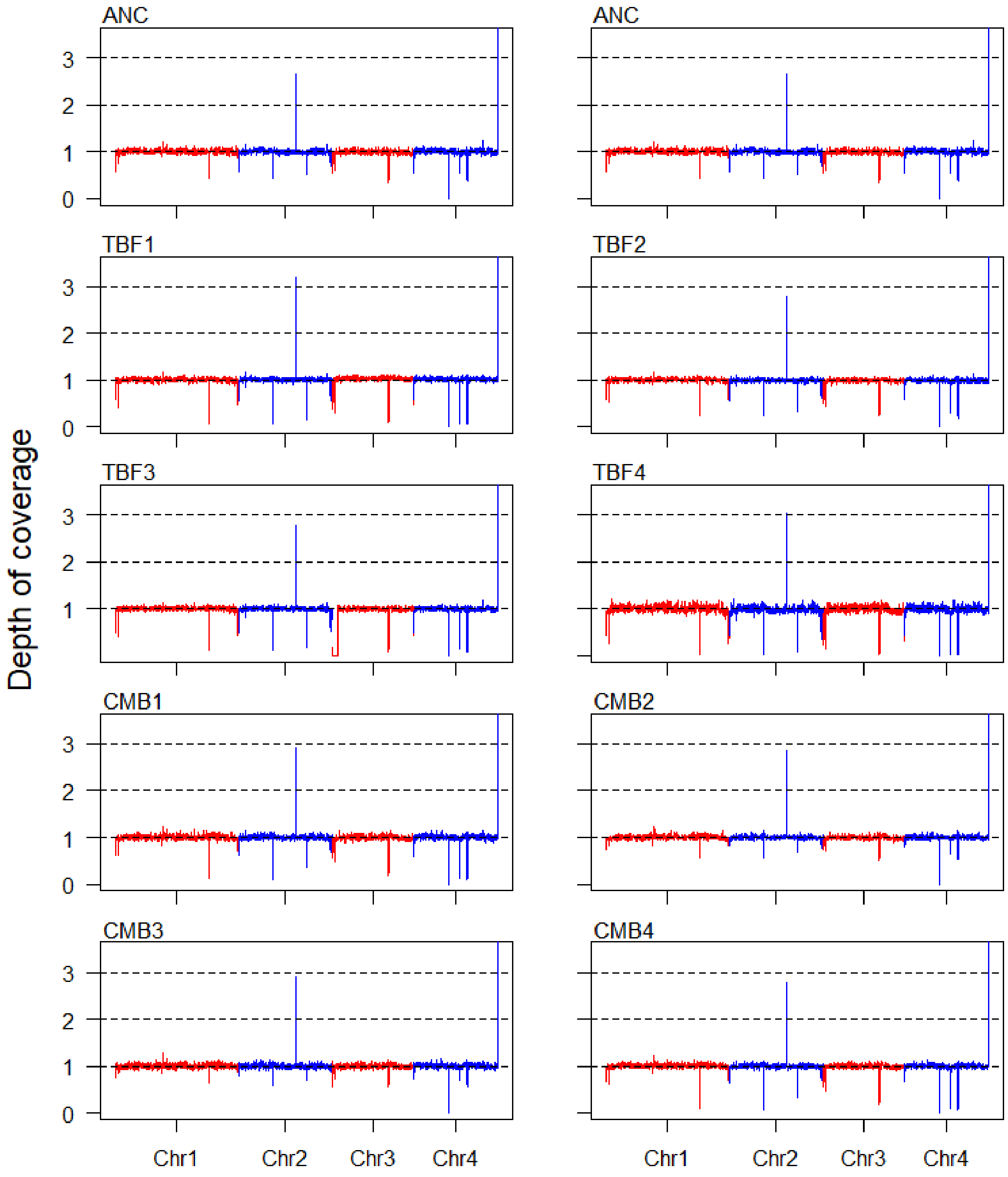

Supporting Data Figure S3 – Profiles of read depth across the genome for the ancestral and each final evolved strain. Chromosomes are shown in alternating colours. Along the y-axis, “1” corresponds to the average read depth; peaks likely correspond to repetitive regions, while troughs likely correspond to regions that are difficult to sequence. The profile for the ancestral strain is shown twice, to facilitate comparison of profiles down each column.

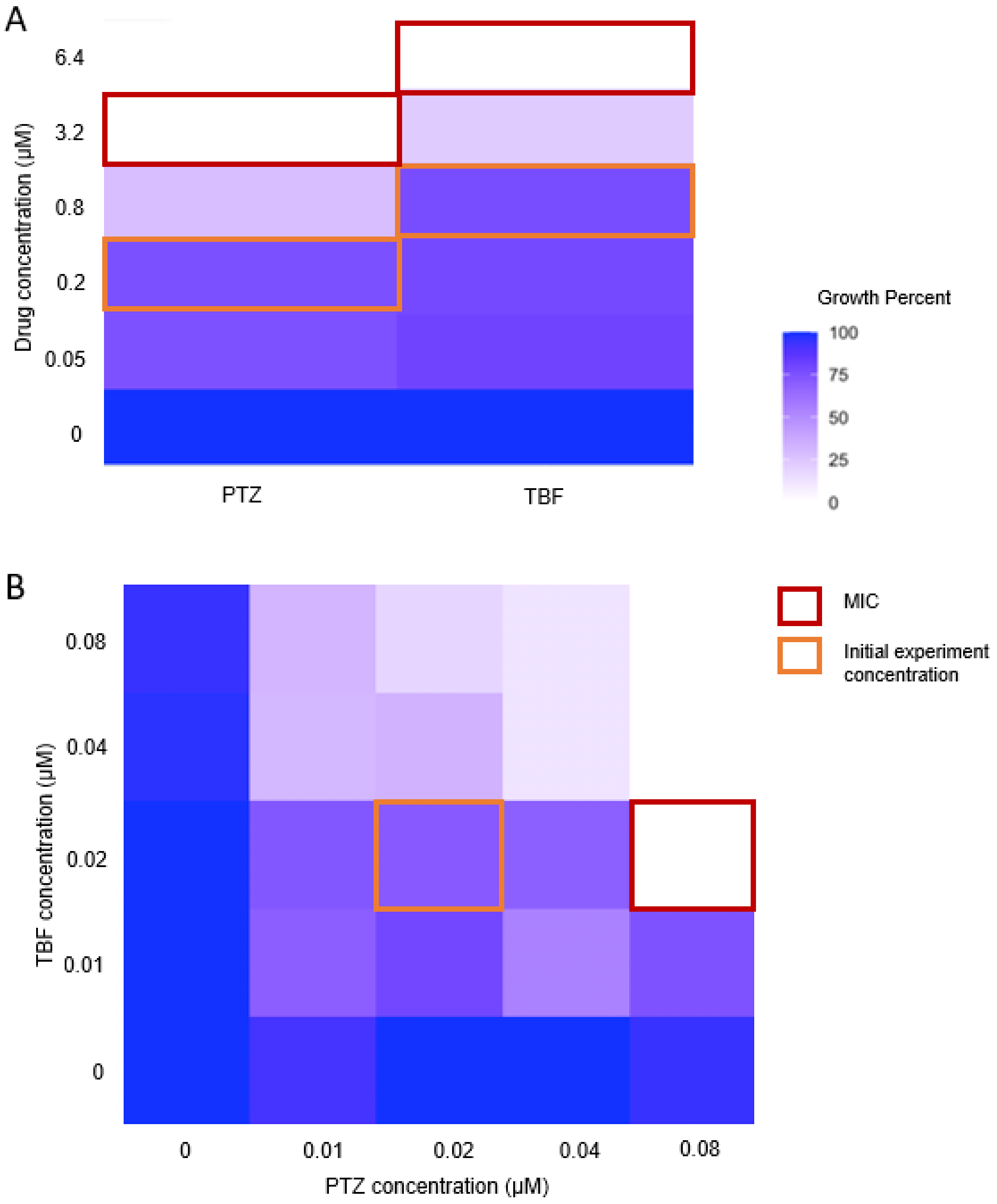

Supporting Data Figure S4 – Impacts of **A)** prothioconazole ("PTZ") or tebuconazole ("TBF"), or **B)** the combination of both fungicides on the growth of *Fusarium graminearum* on solid V8 agar. The minimum inhibitory concentration for each treatment is shown with the red boxes, while the orange boxes indicate the dose that was selected for the initial exposure in the evolution experiment.

Supporting Data Table S1 – Oligos used in this work. (attached file)
